# Sex differences in esophageal cancer overall and by histological subtype

**DOI:** 10.1038/s41598-022-09193-x

**Published:** 2022-03-28

**Authors:** Nickolas Stabellini, Apoorva Krishna Chandar, Amitabh Chak, Amie J. Barda, Mantas Dmukauskas, Kristin Waite, Jill S. Barnholtz-Sloan

**Affiliations:** 1 Graduate Education Office, Case Western Reserve University School of Medicine, Cleveland, OH USA; 2 Department of Hematology-Oncology, University Hospitals Seidman Cancer Center, Breen Pavilion-11100 Euclid Ave, Cleveland, OH 44106 USA; 3 Faculdade Israelita de Ciências da Saúde Albert Einstein, Hospital Israelita Albert Einstein, São Paulo, SP Brazil; 4 Department of Population and Quantitative Health Sciences, Case Western Reserve University School of Medicine, Cleveland, OH USA; 5 Department of Medicine, University Hospitals Cleveland Medical Center, Cleveland, OH USA; 6 Division of Gastroenterology and Liver Disease, University Hospitals Cleveland Medical Center, Case Western Reserve University School of Medicine, Cleveland, OH USA; 7 Department of Pediatrics, University Hospitals Cleveland Medical Center, Cleveland, OH USA; 8 Trans-Divisional Research Program (TDRP), Division of Cancer Epidemiology and Genetics (DCEG), National Cancer Institute, National Institutes of Health, Bethesda, MD USA; 9 Center for Biomedical Informatics and Information Technology (CBIIT), National Cancer Institute, National Institutes of Health, Bethesda, MD USA

**Keywords:** Cancer epidemiology, Gastrointestinal cancer

## Abstract

Esophageal cancer is the seventh most common type of cancer in the world, the sixth leading cause of cancer-related death and its incidence is expected to rise 140% in the world in a period of 10 years until 2025. The overall incidence is higher in males, while data about prognosis and survival are not well established yet. The goal of this study was to carry out a comprehensive analysis of differences between sexes and other covariates in patients diagnosed with primary esophageal cancer. Data from 2005 to 2020 were obtained from the University Hospitals (UH) Seidman Cancer Center and from 2005 to 2018 from SEER. Patients were categorized according to histological subtype and divided according to sex. Pearson Chi-square test was used to compare variables of interest by sex and the influence of sex on survival was assessed by Kaplan Meier, log rank tests and Cox proportional hazards regression models. A total of 1205 patients were used for analysis. Sex differences in all types were found for age at diagnosis, histology, smoking status and prescriptions of NSAIDs and in SCC for age at diagnosis and alcoholism. Survival analysis didn’t showed differences between males and females on univariable and multivariable models. Males have a higher incidence of Esophageal Cancer and its two main subtypes but none of the comprehensive set of variables analyzed showed to be strongly or unique correlated with this sex difference in incidence nor are they associated with a sex difference in survival.

## Introduction

Esophageal cancer (EC) is the seventh most common type of cancer in the world and the sixth leading cause of cancer-related death, with a 5-year survival rate of 15–20%^1,2^. Its incidence is expected to rise 140% in the world in a period of 10 years until 2025^3^. In the United States, it is estimated that, in 2021, there will be 19,260 new cases (15,310 in males and 3959 in females; with an ~ fourfold higher incidence in males) and 15,530 deaths (12,410 in males and 3120 in females; with an ~ fourfold higher death rates in males) from esophageal cancer^4^. Strikingly, the epidemiology in the western world has changed during the last 4 decades with a sharp decline in the proportion of squamous cell carcinomas (SCC) and an increase in the proportion of adenocarcinomas^5^.

Esophageal adenocarcinomas (EACs) and SCCs differ mainly in terms of tumor location and by their predisposing factors. Barrett’s esophagus (BE) is the only known pre-malignant precursor to EAC, and virtually all EAC is said to arise in a background of BE^6^. Smoking and alcohol are the main risk factors for SCC, and these two risk factors seem to confer a synergistic risk effect^7–9^. EACs are associated with GERD (Gastroesophageal reflux disease), central obesity and smoking but not alcohol^9^. Smoking is a stronger risk factor of SCC with approximately sixfold odds compared to twofold odds for EAC^10,11^. Esophageal SCC can be present throughout the middle esophagus, while EAC can be present throughout the distal esophagus^12^. Treatment depends on the location and the histological subtype, and may be endoscopic for very early asymptomatic disease, only surgical for localized disease, multimodal for advanced disease and palliative non-surgical for metastatic disease^1,13–15^.

As in other types of cancer, sex differences in incidence are also seen in esophageal cancer. In the United States, 76% of cases of adenocarcinoma from 1973 to 2012 occurred in white males^16,17^. It is estimated that the odds for EAC is 7–10 times greater and the odds for SCC is 3–4 times greater in males than females^18^. Also, sex has been shown to be an independent prognostic marker in SCC but not in EAC, with females having better survival^19–22^. In addition, there is a report of greater regional recurrence and distant metastasis in males when compared to females, indicating that there is greater control of the disease after radiotherapy in females^19^.

Despite published data showing sex differences for esophageal cancer, root causes are still poorly understood. To our knowledge, no studies have analyzed sex differences across a large spectrum of variables in both SCC and EAC. We hypothesized that there may be differences in epidemiological criteria, risk factors or treatment patterns that explain the sex differences in incidence and outcomes. Therefore, we carried out a comprehensive analysis on solid databases of differences between sexes and other covariates in patients diagnosed with primary esophageal cancer.

## Methods

Data were obtained from the University Hospitals (UH) Seidman Cancer Center research data repository consisting of patient records from 2005 to 2020. The Data repository is based on CAISIS, an open source web-based cancer data management system that integrates research with patient care and has integration from disparate sources (Soarian, NGS Labs, Sunrise Clinical Manager, Tumor Registry, Via Oncology, OnCore, MosiaQ, PRO tools and others) to provide comprehensive data on the UH Seidman cancer patient population^23^. Patient records were deidentified and all the analysis were performed in accordance with relevant guidelines and regulations, respecting the Declaration of Helsinki. The study with the waiver of the informed consent was approved by the University Hospitals of Cleveland Institutional Review Board (IRB).

**Figure 1:**
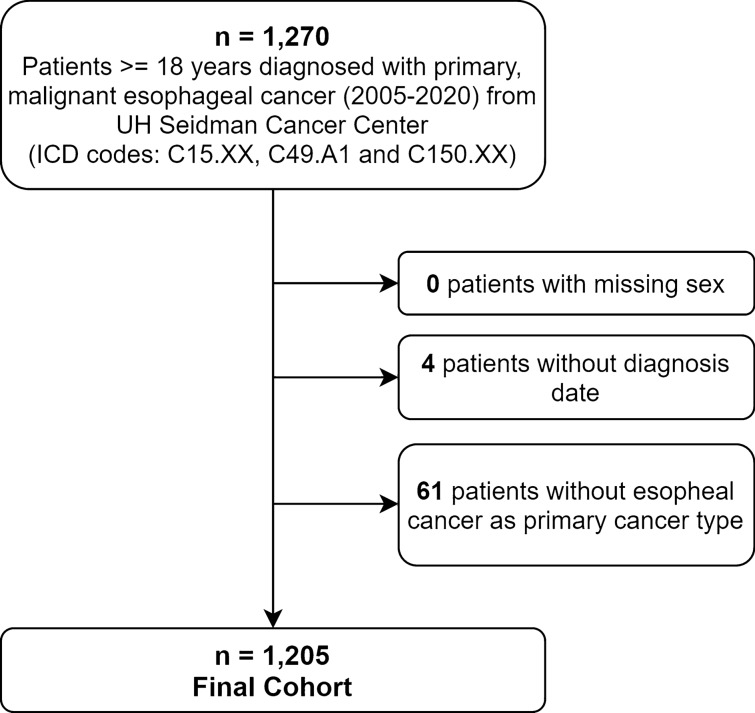
Cohort description with inclusion and exclusion criteria for UH institutional database. The final cohort included 1205 patients diagnosed with esophageal cancer from 2005 to 2020 with ≥ 18 years, excluding those without unknown diagnosis date, missing gender and without primary esophageal cancer).

The initial cohort included patients ≥ 18 years old who were diagnosed with primary malignant esophageal cancer between 2005 and 2020 (ICD codes C15.XX, C49.A1 and 150.XX)^24,25^. Patients were excluded from analysis if they had missing sex information, unknown date of diagnosis or a prior history of cancer. The cohort selection for analysis is described in Fig. 1.

Data extracted from the UH platform for each patient included basic demographics (such as age at diagnosis, sex, race, etc.), comorbidities, histology/subtype, staging, laboratory results, vital signs, medications, and cancer treatment information (chemotherapy, hormone therapy, immunotherapy, surgery, radiation). Treatment information was only included if it was related to esophageal cancer or the anatomical location of the esophagus. From the list of medications, we selected the drugs and classes commonly used on treatment for esophageal cancer or those drugs that can be risk factors^26^. Patients with a recorded date of death obtained from the EMR and state records were considered deceased.

Our final analysis included 29 categorical variables, grouped into General Characteristics, Cancer Characteristics, Risk Factors and General Treatment. General Characteristics variables included age at diagnosis, sex, median income, race and ethnicity. Cancer Characteristics variables included histological subtype, clinical stage and pathological stage. Risk Factors included Charlson comorbidity score^27^, smoking status and presence/absence of the following comorbidities: obesity, BE, alcoholism, achalasia, previous gastrectomy, gastritis, gastroesophageal reflux, H.pylori infection and long term use of NSAIDs. General treatment variables included whether the patient received the following therapies: chemotherapy, immunotherapy, radiation (of the esophagus or nearby anatomical area), surgery of the esophagus, Cisplatin, Fluorouracil, Paclitaxel, H2 antagonists, proton pump inhibitors (PPIs), NSAIDs and statins. Time to treatment variables (time to chemotherapy, time to radiation, time of radiation and time to surgery) were calculated for those which received the respective types of treatment according to last day and first day registered on the EMR, surgery date, diagnosis date and categorized as < 40 days and ≥ 40 days.

Age was categorized based on epidemiology reports and clinical experience as ≥ 18–55 years, 56–70 years and > 70 years^38,28,29^. Race was categorized as white, black, or other. Ethnicity was categorized as Hispanic, non-Hispanic or other. Estimated median income was determined by the patient’s zip code and categorized as < $43,235, $43,235-$64,446, or > $64,446 (25%, 50% and 75% percentiles). The risk factors were selected from the list of comorbidities of each patient based on those most related to esophageal cancer according to the literature and clinical experience^30,31^. Histological subtype was categorized into Squamous Cell Carcinoma—SCC (ICD-O-3 8050-8084) or Esophageal Adenocarcinoma—EAC (ICD-O-3 8140-8384)^32^. The categorization process is summarized in supplementary Table S1.

SEER data was used to compare and validate our findings with the general population. Data were obtained from SEER*stat software based on SEER Research Plus Database for esophageal cancer diagnosis between 2005 and 2018^33^. The variables analyzed were categorized following the methodology applied to the UH Database and included sex, age at diagnosis, race, ethnicity, histology, staging, chemotherapy, radiotherapy, surgery, vital status and median survival.

The sample was divided according to sex as male or female. Pearson Chi-Square test was used to compare variables of interest by sex, disregarding patients with missing values, with p < 0.05 being considered significant. The influence of sex on survival was first assessed using Kaplan Meier analysis generating median survival by sex with 95% confidence intervals (95% CI) and log rank tests by sex. Cox proportional hazards regression models were used after getting the assumptions checked to assess univariable and multivariable models of overall survival by sex and by sex and histological subtype (EAC and SCC). The variables selected for the multivariable model overall and by histological subtype were those with p < 0.20 in the univariable model and those with clinical importance. Correlated variables checked by chi-square test were not included in the final model. All analyses were performed using RStudio 1.2.1335 software^34^.

## Results

### All types of esophageal cancer

**Figure 2:**
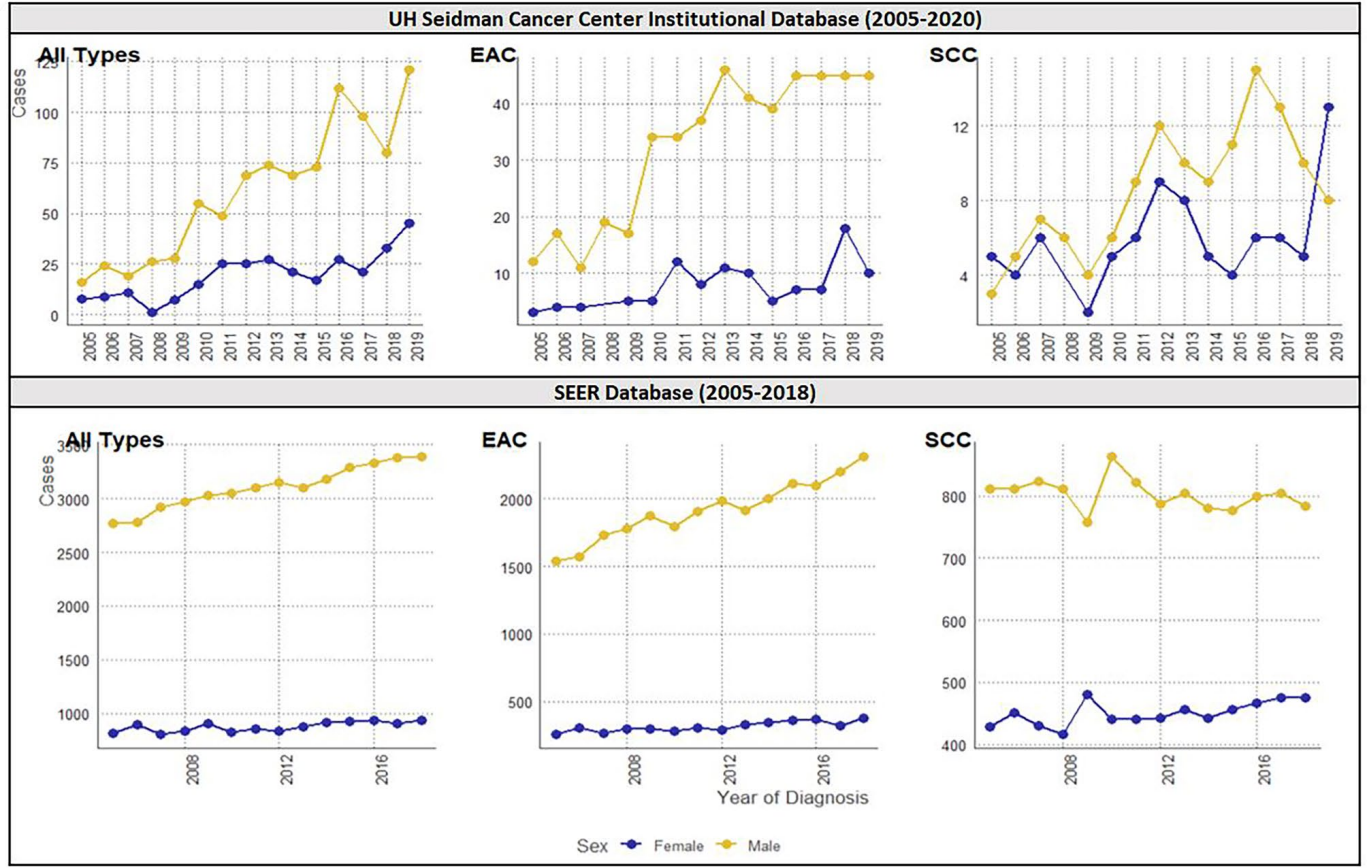
Plots of cases by year from UH Seidman Cancer Center (2005–2020) and SEER (2005–2018) for all types of esophageal cancer, esophageal adenocarcinoma (EAC) and squamous cell carcinoma (SCC). All types of esophageal cancer cases are increasing in the US.

Using data from years 2005 to 2020 we analyzed a total of 1205 patients for all types of esophageal cancer, with 75.8% (913) males and 24.2% (292) females, establishing a male: female ratio of about of 3:1. The evolution of cases by year is shown on Fig. 2. For general characteristics (Table 1), sex differences existed only for age at diagnosis (p < 0.001), with a predominance of females > 70 years old (46.9% of females) and males between 56 and 70 years old (48.2% of males). There were no significant differences for median income (p = 0.12), race (p = 0.06) and ethnicity (p = 0.21). When cancer characteristics (Table 2), we found a difference for histology (p < 0.001) with a predominance of EAC in both groups (79.2% in males and 56.5% in females) and no significant differences were found for clinical staging (p = 0.21) and pathological staging (p = 0.08).

**Table 1:** General characteristics by sex for all types of esophageal cancer combined, esophageal adenocarcinoma (EAC) and squamous cell carcinoma (SCC), UH Seidman Cancer Center Database (2005–2020).

	All types (n = 1205)			EAC (n = 596)			SCC (n = 212)		
	Male	Female	p value	Male	Female	p value	Male	Female	p value
	913 (75.8%)	292 (24.2%)		487 (81.7%)	109 (18.3%)		128 (60.4%)	84 (39.6%)	
**Age at diagnosis—n (%)**									
18-55y	146 (16%)	48 (16.4%)	< 0.001^a^	83 (17%)	14 (12.8%)	0.16^a^	21 (16.4%)	17 (20.2%)	0.02^a^
56-70y	440 (48.2%)	107 (36.6%)		235 (48.3%)	47 (43.1%)		73 (57%)	31 (36.9%)	
> 70y	327 (35.8%)	137 (46.9%)		169 (34.7%)	48 (44%)		34 (26.6%)	36 (42.9%)	
**Median income—n (%)**									
< $43,235	155 (23.9%)	58 (28.9%)	0.12^a^	80 (19.7%)	20 (22%)	0.58^a^	46 (41.4%)	25 (39.1%)	0.94^a^
$43,235-$64,446	350 (53.9%)	92 (45.8%)		230 (56.5%)	46 (50.5%)		41 (36.9%)	24 (37.5%)	
> $64,446	144 (22.2%)	51 (25.4%)		97 (23.8%)	25 (27.5%)		24 (21.6%)	15 (23.4%)	
Unknown	264	91		80	18		17	20	
**Race—n (%)**									
White	672 (81.9%)	195 (75.3%)	0.06^a^	358 (83.8%)	73 (80.2%)	0.06^a^	57 (52.8%)	37 (52.9%)	0.98^a^
Black	66 (8%)	29 (11.2%)		14 (3.3%)	0		32 (29.6%)	20 (28.6%)	
Other	83 (10.1%)	35 (13.5%)		55 (12.9%)	18 (19.8%)		19 (17.6%)	13 (18.6%)	
Unknown	92	33		60	18		20	14	
**Ethnicity—n (%)**									
Hispanic	9 (1.1%)	3 (1.1%)	0.21^a^	4 (0.9%)	1 (1%)	1^a^	2 (1.6%)	0	0.67^a^
Non-hispanic	845 (98.9%)	27.3% (98.6%)		451 (99.1%)	103 (99%)		122 (98.4%)	80 (100%)	
Other	0	1 (0.4%)		0	0		0	0	
Unknown	59	15		32	5		4	4	

^a^Chi-Square test.

**Table 2:** Cancer characteristics by sex for all types of esophageal cancer combined, esophageal adenocarcinoma (EAC) and squamous cell carcinoma (SCC), UH Seidman Cancer Center Database (2005–2020).

	All types (n = 1205)					
	Male	Female	p value			
	913 (75.8%)	292 (24.2%)				
**Histology—n (%)**			< 0.001^a^			
Adenocarcinoma (EAC)	487 (79.2%)	109 (56.5%)				
Squamous cell carcinoma (SCC)	128 (20.8%)	84 (43.5%)				
Unknown	298	99				

	Clinical staging—n (%)			Pathological staging—n (%)		
	Male	Female	p value	Male	Female	p value
0	1 (0.3%)	2 (1.9%)	0.21^a^	3 (1.7%)	3 (6.4%)	0.08^a^
I	33 (9.4%)	11 (10.4%)		47 (26.3%)	19 (40.4%)	
II	54 (15.4%)	19 (17.9%)		51 (28.5%)	10 (21.3%)	
III	121 (34.5%)	41 (38.7%)		43 (24%)	10 (21.3%)	
IV	142 (40.5%)	33 (31.1%)		35 (19.6%)	5 (10.6%)	
Unknown	562	186		734	245	
0	0	0	0.81^a^	3 (2.1%)	0	0.63^a^
I	29 (11.7%)	5 (10.6%)		42 (29%)	11 (42.3%)	
II	34 (13.8%)	6 (12.8%)		38 (26.2%)	5 (19.2%)	
III	87 (35.2%)	20 (42.6%)		32 (22.1%)	6 (23.1%)	
IV	97 (39.3%)	16 (34%)		30 (20.7%)	4 (15.4%)	
Unknown	240	62		342	83	
0	1 (1.5%)	1 (2%)	0.57^a^	0	2 (12.5%)	0.17^a^
I	1 (1.5%)	3 (6.1%)		3 (17.6%)	5 (31.2%)	
II	14 (21.2%)	12 (24.5%)		8 (47.1%)	5 (31.2%)	
III	24 (36.4%)	19 (38.8%)		3 (17.6%)	4 (25%)	
IV	26 (39.4%)	14 (28.6%)		3 (17.6%)	0	
Unknown	62	35		111	68	

^a^Chi-square test.

There was a difference for risk factors (Table 3) in smoking status (p = 0.01) with a predominance of former smokers in males overall and by histological subtype (58.1% in males and 45% in females overall), with no difference for Charlson Score (p = 0.28), obesity (p = 0.11), BE (p = 0.22), alcoholism (p = 0.35), achalasia (p = 0.64), previous gastrectomy (p = 0.17), gastritis (p = 0.28), gastroesophageal reflux (p = 0.42), H.pylori infection (p = 0.80) and long term use of NSAIDs (p = 0.96).

**Table 3:** Risk factors by sex for all types of esophageal cancer combined, esophageal adenocarcinoma (EAC) and squamous cell carcinoma (SCC), UH Seidman Cancer Center Database (2005–2020).

	All types (n = 1205)			EAC (n = 596)			SCC (n = 212)		
	Male	Female	p value	Male	Female	p value	Male	Female	p value
	913 (75.8%)	292 (24.2%)		487 (81.7%)	109 (18.3%)		128 (60.4%)	84 (39.6%)	
**Charlson comorbidity score—n (%)**									
0	1 (0.1%)	0	0.28^a^	0	0	0.30^a^	0	0	0.58^a^
1 to 2	558 (61.1%)	182 (62.3%)		305 (62.6%)	67 (61.5%)		79 (61.7%)	53 (63.1%)	
3 to 4	248 (27.2%)	87 (29.8%)		136 (27.9%)	36 (33%)		31 (24.2%)	23 (27.4%)	
> = 5	106 (11.6%)	23 (7.9%)		46 (9.4%)	6 (5.5%)		18 (14.1%)	8 (9.5%)	
**Smoking status – n (%)**									
Yes	75 (15.6%)	25 (17.9%)	0.01^a^	34 (13.9%)	5 (9.85)	0.05^a^	19 (27.5%)	9 (23.1%)	0.49^a^
No	126 (26.2%)	52 (37.1%)		69 (28.2%)	23 (45.1%)		7 (10.1%)	7 (17.9%)	
Former	279 (58.1%)	63 (45%)		142 (58%)	23 (45.1%)		43 (62.3%)	23 (59%)	
Unknown	433	152		242	58		59	45	
**Additional known risk factors—n (%)**									
Obesity	115 (12.6%)	48 (16.4%)	0.11^a^	76 (15.6%)	23 (21.1%)	0.21^a^	6 (4.7%)	4 (4.8%)	1^a^
Barrett's Esophagus	158 (17.3%)	41 (14%)	0.22	109 (22.4%)	23 (21.1%)	0.87^a^	6 (4.7%)	5 (6%)	0.92^a^
Alcoholism	111 (12.2%)	29 (9.9%)	0.35^a^	39 (8%)	4 (3.7%)	0.16^a^	47 (36.7%)	19 (22.6%)	0.04^a^
Achalasia	5 (0.5%)	3 (1%)	0.64^a^	1 (0.2%)	1 (0.9%)	0.80^a^	1 (0.8%)	0	1a
Previous Gastrectomy	17 (1.9%)	10 (3.4%)	0.17^a^	15 (3.1%)	5 (4.6%)	0.62^a^	1 (0.8%)	4 (4.8%)	0.15^a^
Gastritis	107 (11.7%)	27 (9.2%)	0.28a	51 (10.5%)	9 (8.3%)	0.60^a^	12 (9.4%)	7 (8.2%)	0.98^a^
Gastroesophageal Reflux	452 (49.5%)	153 (52.4%)	0.42^a^	258 (53%)	62 (56.9%)	0.52^a^	59 (46.1%)	43 (51.2%)	0.55^a^
H.Pylori Infection	6 (0.7%)	3 (0.1%)	0.80^a^	2 (0.4%)	0	1^a^	2 (1.6%)	2 (2.4%)	1^a^
Long-term use of NSAIDs	4 (0.4%)	2 (0.7%)	0.96^a^	3 (0.6%)	1 (0.9%)	1^a^	0	1 (1.2%)	0.83^a^

^a^Chi-square test.

**Figure 3:**
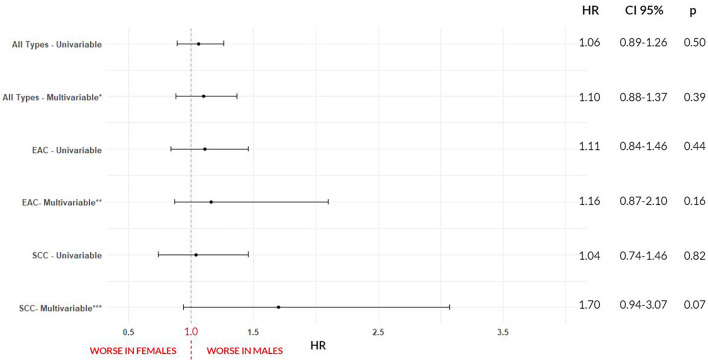
Forest plot of sex differences in survival for esophageal cancer (all types), esophageal adenocarcinoma (EAC) and squamous cell carcinoma (SCC). Univariable and multivariable cox models represented, UH Seidman Cancer Center Database (2005–2020). *Adjusted for: age at diagnosis, race, ethnicity, histology, obesity, Barret’s, gastrectomy, gastritis, gastroesophageal reflux, chemotherapy, surgery and radiotherapy. **Adjusted for: age at diagnosis, smoking status, obesity, Barret’s, alcoholism, achalasia, gastrectomy, gastritis, gastroesophageal reflux, h.pilory, chemotherapy, surgery and radiotherapy. ***Adjusted for: race, Barret’s, gastritis, gastroesophageal reflux, h.pilory, radiotherapy, surgery, smoking status and alcoholism.

**Figure 4:**
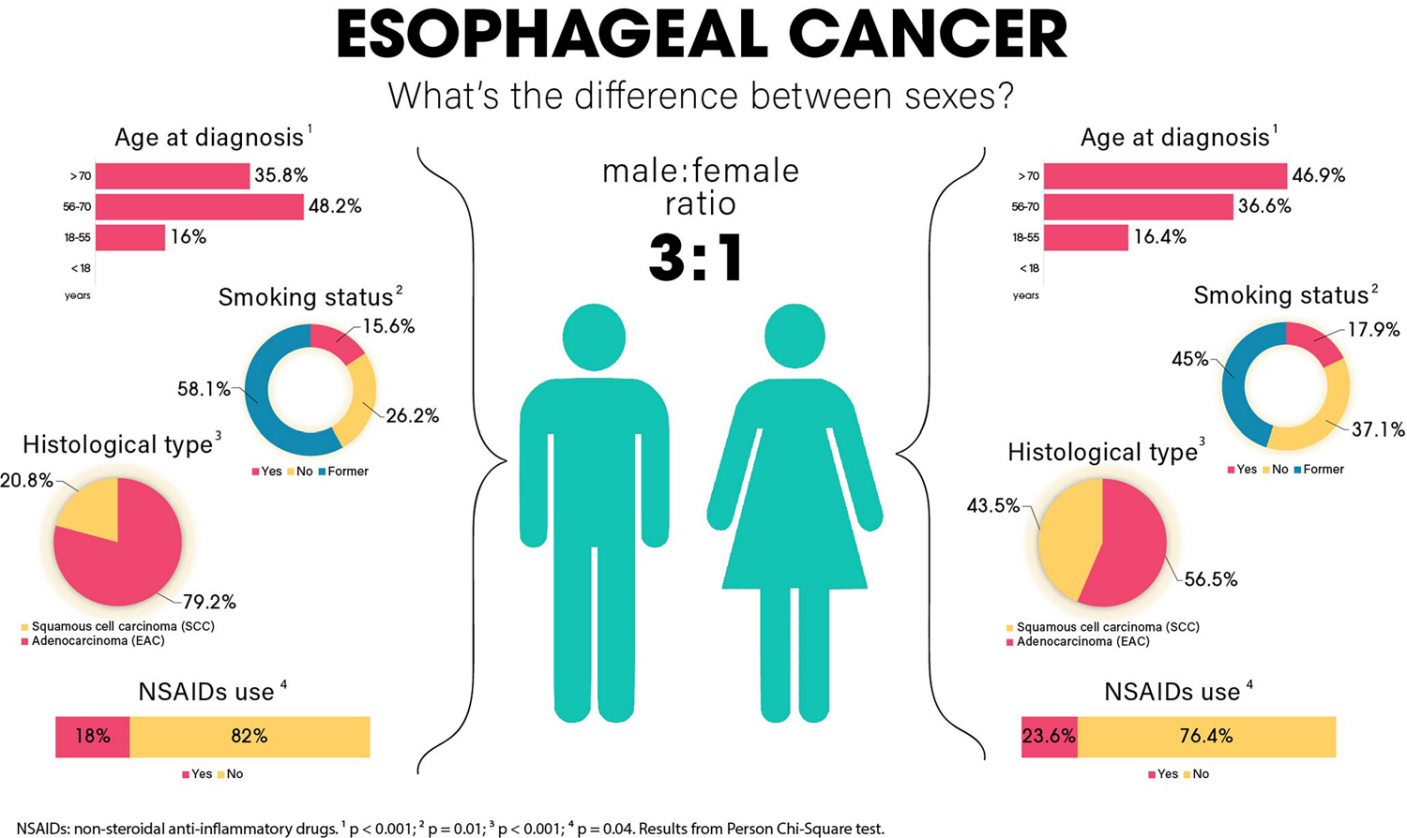
Sex differences for esophageal cancer overall. Higher in males (3:1 ratio) and statistical differences for age at diagnosis (p < 0.001), smoking status (p = 0.01), histological subtype (p < 0.001) and NSAIDs use (p = 0.04), UH Seidman Cancer Center Database (2005–2020).

For treatment characteristic (Table 4), differences were observed in the prescription of NSAIDs (p = 0.04). No difference were seen for chemotherapy (p = 0.82), immunotherapy (p = 0.13), radiotherapy (p = 0.50), surgery (p = 0.17), time to chemotherapy (p = 0.77), time to radiation (p = 1.00), time of radiotherapy (p = 0.72), time to surgery (p = 0.93), Cisplatin use (p = 0.97), Fluorouracil use (p = 0.23), Paclitaxel use (p = 1.00), H2 antagonists use (p = 0.52), PPIs use (p = 0.96) and Statins use (p = 0.93).The median survival was 27 months for males vs 32 for females (p = 0.50) (Table 5). The univariable model did not show inferiority for males (HR = 1.06, CI 0.89–1.26, p = 0.50). The multivariable model included the statistically significant variables (p < 0.20) age at diagnosis, race, ethnicity, histology, obesity, BE, gastrectomy, gastritis, gastroesophageal reflux, chemotherapy and surgery. Pathological stage, although statistically significant, was not included in the model due to the high number of unknown values. Radiotherapy was included despite p > 0.20 due to reports in the literature of better survival in females after this type of treatment^19^. The multivariable model also did not show inferiority for males (HR = 1.10, CI 0.88–1.27, p = 1.37). Both models are summarized on Fig. 3. Sex Differences for all types of esophageal cancer combined are summarized on Fig. 4.

**Table 4:** General treatment by sex for all types of esophageal cancer combined, esophageal adenocarcinoma (EAC) and squamous cell carcinoma (SCC), UH Seidman Cancer Center Database (2005–2020).

	All types (n = 1205)			EAC (n = 596)			SCC (n = 212)		
	Male	Female	p value	Male	Female	p value	Male	Female	p value
	913 (75.8%)	292 (24.2%)		487 (81.7%)	109 (18.3%)		128 (60.4%)	84 (39.6%)	
**Type of treatment—n (%)**									
Chemotherapy	484 (53%)	152 (52.1%)	0.82^a^	345 (70.8%)	76 (69.7%)	0.90^a^	93 (72.7%)	61 (72.6%)	1^a^
Immunotherapy	42 (4.6%)	7 (2.4%)	0.13^a^	36 (7.4%)	5 (4.6%)	0.40^a^	3 (2.3%)	1 (1.2%)	0.93^a^
Radiotherapy	145 (15.9%)	41 (14%)	0.50^a^	99 (20.3%)	23 (21.1%)	0.96^a^	22 (17.2%)	10 (11.9%)	0.39^a^
Surgery	403 (44.1%)	115 (39.4%)	0.17^a^	264 (54.2%)	58 (53.2%)	0.93^a^	57 (44.5%)	32 (38.1%)	0.43^a^
**Time to chemotherapy—n (%)**									
< 40 days	192 (54.5%)	59 (56.7%)	0.77^a^	141 (53.6%)	31 (56.4%)	0.82^a^	32 (54.2%)	23 (56.1%)	1^a^
> = 40 days	160 (45.5%)	45 (43.3%)		122 (46.4%)	24 (43.6%)		27 (45.8%)	18 (43.9%)	
**Time to radiotherapy—n (%)**									
< 40 days	65 (52%)	19 (51.4%)	1^a^	46 (54.1%)	9 (45%)	0.62^a^	8 (38.1%)	5 (50%)	0.81^a^
> = 40 days	60 (48%)	18 (48.6%)		39 (45.9%)	11 (55%)		13 (61.9%)	5 (50%)	
**Time of radiotherapy—n (%)**									
< 40 days	76 (58.9%)	23 (63.9%)	0.72^a^	48 (52.7%)	10 (50%)	1^a^	15 (78.9%)	7 (77.8%)	1^a^
> = 40 days	53 (41.1%)	13 (36.1%)		43 (47.3%)	10 (50%)		4 (21.1%)	2 (22.2%)	
**Time to surgery—n (%)**									
< 40 days	59 (24%)	19 (25.3%)	0.93^a^	38 (21.1%)	11 (28.9%)	0.40^a^	9 (24.3%)	6 (24%)	1^a^
> = 40 days	187 (76%)	56 (74.7%)		142 (78.9%)	27 (71.1%)		28 (75.7%)	19 (76%)	
**Medications prescribed—n (%)**									
Cisplatin	60 (6.6%)	20 (6.8%)	0.97^a^	33 (6.8%)	6 (5.5%)	0.78^a^	11 (8.6%)	12 (14.3%)	0.28^a^
Fluorouracil	130 (14.2%)	33 (11.3%)	0.23^a^	88 (18.1%)	23 (21.1%)	0.54^a^	10 (7.8%)	5 (6%)	0.80^a^
Paclitaxel	175 (19.2%)	56 (19.2%)	1^a^	108 (22.2%)	29 (26.6%)	0.38^a^	31 (24.2%)	15 (17.9%)	0.35^a^
H2 Antagonists	231 (25.3%)	80 (27.4%)	0.52^a^	134 (27.5%)	37 (33.9%)	0.22^a^	38 (29.7%)	23 (27.4%)	0.85^a^
PPIs	338 (37%)	107 (36.6%)	0.96^a^	176 (36.1%)	42 (38.5%)	0.71^a^	62 (48.4%)	40 (47.6%)	1^a^
NSAIDs	164 (18%)	69 (23.6%)	0.04^a^	79 (16.2%)	25 (22.9%)	0.12^a^	41 (32%)	27 (32.1%)	1^a^
Statins	129 (14.1%)	40 (13.7%)	0.93^a^	61 (12.5%)	17 (15.6%)	0.48^a^	23 (18%)	15 (17.9%)	1^a^

^a^Chi-square test.

**Table 5:** Median survival by sex for all types of esophageal cancer combined, esophageal adenocarcinoma (EAC) and squamous cell carcinoma (SCC), UH Seidman Cancer Center Database (2005–2020).

	All types (n = 1205)			EAC (n = 596)			SCC (n = 212)		
	Male	Female	p value	Male	Female	p value	Male	Female	p value
	913 (75.8%)	292 (24.2%)		487 (81.7%)	109 (18.3%)		128 (60.4%)	84 (39.6%)	
**Vital status—n (%)**									
Alive	380 (41.6%)	131 (44.9%)	0.36^a^	188 (38.6%)	49 (45%)	0.26^a^	43 (33.6%)	29 (34.5%)	1^a^
Dead	533 (58.4%)	161 (55.1%)		299 (61.4%)	60 (55%)		85 (66.4%)	55 (65.5%)	
**Median survival—months (CI 95%)**									
	27 (24–36)	32 (23–53)	0.50^b^	27 (24–38)	29 (18-NA)	0.40^b^	17 (12–26)	25 (13–41)	0.80^b^

^a^Chi-square test.

^b^Log-rank test.

### Esophageal adenocarcinoma (EAC)

Of the total cohort of 1205 patients, 596 (49.46%) had a histological classification of EAC, with 487 males (81.7%) and 109 females (18.3%), establishing a male: female ratio of about 4:1. The evolution of cases by year with EAC is shown on Fig. 2. In General Characteristics for EAC (Table 1) there was no difference in age at diagnosis (p = 0.16), median income (p = 0.58), race (p = 0.06) and ethnicity (p = 1.00). In males there was a predominance of the categories 56–70 years (48.3%), $43,235–$64,446 (53.9%), white race (83.9%) and non-Hispanic (99.1%). In females predominated the categories > 70 years (44%), $43,235–$64,446 (50.5%), white race (80.2%) and non-Hispanic (99%). The white: black ratio was of 30:1. In cancer characteristics for EAC (Table 2), there was no difference in clinical staging (p = 0.81) and in pathological staging (p = 0.63). Clinical staging IV predominated in males (39.3%) and clinical staging III in females (42.6%). Pathological staging I predominated in both groups (29% in males and 42.3% in females).

**Figure 5:**
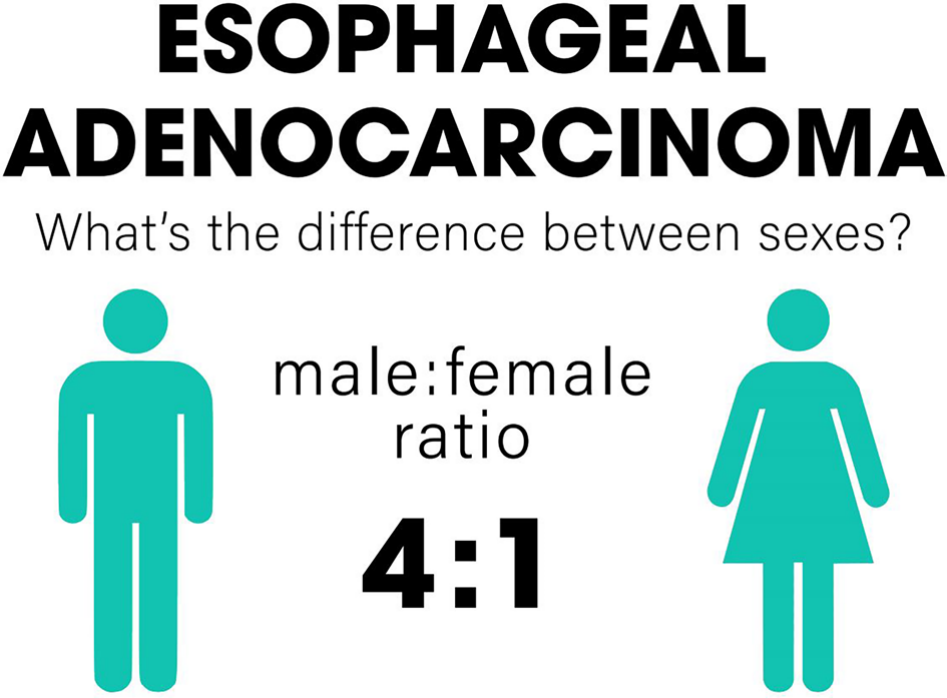
Sex differences for esophageal adenocarcinoma (EAC). Higher in males (ratio 4:1), UH Seidman Cancer Center Database (2005–2020).

For risk factors for EAC (Table 3) there were no differences in Charlson Score (p = 0,30), smoking status (p = 0.05), obesity (p = 0.21), BE (p = 0.87), alcoholism (p = 0.16), achalasia (p = 0.80), previous gastrectomy (p = 0.62), gastritis (p = 0.60), gastroesophageal reflux (p = 0.52), H.pylori infection (p = 1.00) and long-term use of NSAIDs (p = 1.00). In treatment characteristics for EAC (Table 4) there was no difference in chemotherapy (p = 0.90), immunotherapy (p = 0.40), radiotherapy (p = 0.96), surgery (p = 0.93), time to chemotherapy (p = 0,82), time to radiation (p = 0.62), time of radiation (p = 1.00), time to surgery (p = 0.40), Cisplatin use (p = 0.78), Fluorouracil use (p = 0.54), Paclitaxel use (p = 0.38), H2 antagonists use (p = 0.22), PPIs use (p = 0.71), NSAIDs use (p = 0.12) and Statins use (p = 0.48). For EAC only, the median survival was 27 months for males and 29 for females (p = 0.40) (Table 5). The univariable model did not show inferiority for males (HR = 1.11, CI 0.84–1.46, p = 0.44). For the multivariable model were selected the statistically significant variables (p < 0.20) age at diagnosis, smoking status, obesity, BE, alcoholism, achalasia, gastrectomy, gastritis, gastroesophageal reflux, H.pylori, chemotherapy and surgery. Pathological Stage was not included in the model despite p < 0.20 due to the high number of unknown values. Radiotherapy was included in the model despite p > 0.20 due to reports of greater survival in females after this type of treatment^19^. This model also did not show inferiority for males (HR = 1.16 CI 0.87–2.10, p = 0.16). The models are summarized on Fig. 3. Sex differences for EAC are summarized on Fig. 5.

### Squamous cell carcinoma (SCC)

Of the total cohort of 1205 patients, 212 (17.59%) had a histological classification of SCC, with 128 males (60.4%) and 84 females (39.6%), establishing a male:female ratio of about 3:2. The evolution of cases with SCC by year is shown on Fig. 2. In General Characteristics for SCC (Table 1), there was a difference for age at diagnosis (p = 0.02), with a predominance of > 70 years in females (42.9%) and 57–70 years in males (57%). There were no differences in median income (p = 0.94), race (p = 0.98) and ethnicity (p = 0.67). For median income, there was a predominance of $43,235–$64,446 for both groups (36.9% in males and 37.5% in females). For race, there was white predominance in both males (52.8%) and females (52.9%), establishing a white: black ratio of 2:1. For ethnicity there was a predominance of non-Hispanics (98.4% in males and 100% in females). For cancer characteristics for SCC (Table 2), there was no difference in clinical staging (p = 0.57) or in pathological staging (p = 0.17). In males, clinical staging IV (39.4%) and pathological staging II (47.1%) predominated. In females, clinical staging III (38.8%) and pathological staging I and II (31.2% for each) predominated.

**Figure 6:**
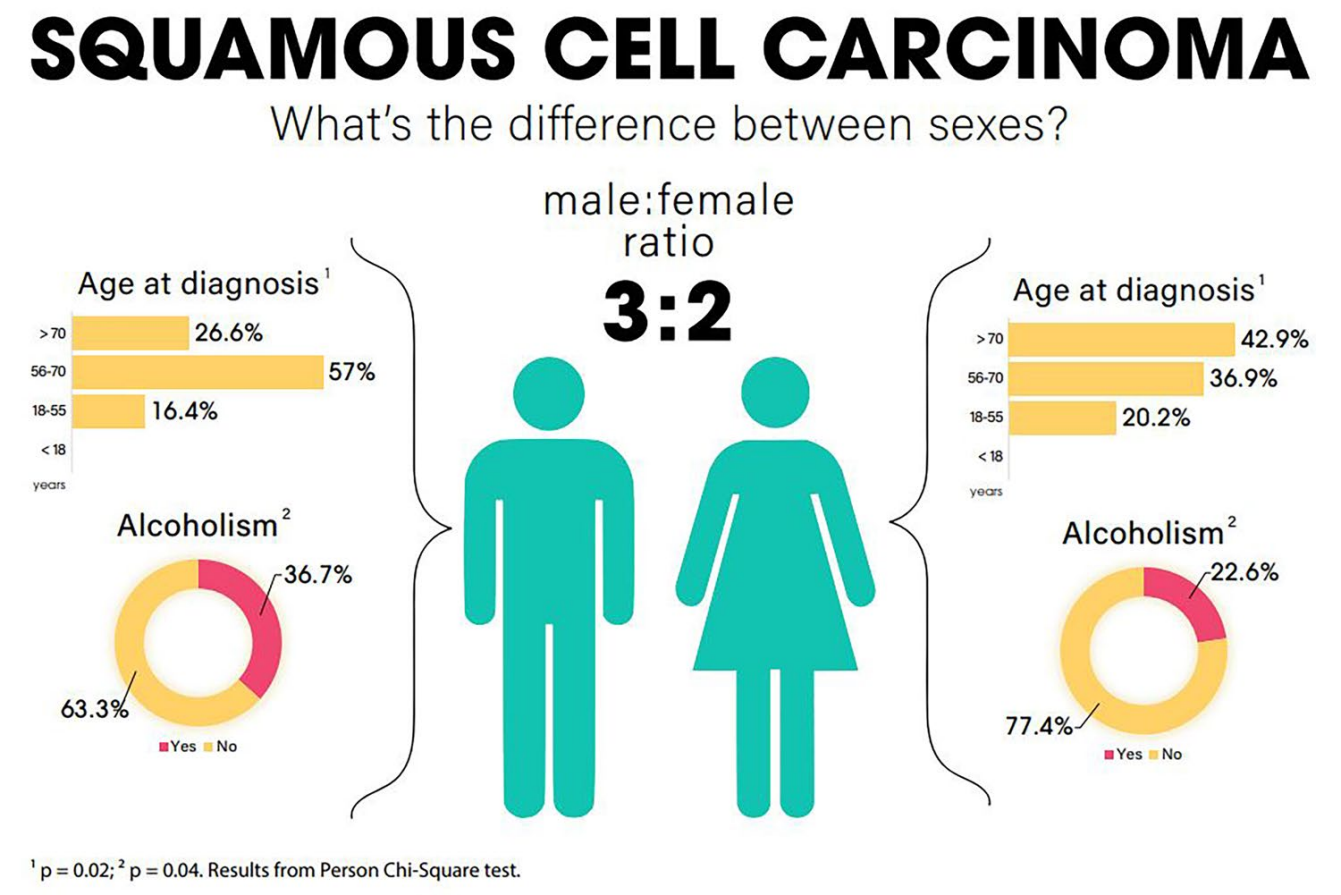
Sex differences for squamous cell carcinoma (SCC). Higher in males (ratio 3:2) and statistical differences in age a diagnosis (p = 0.02) and alcoholism (p = 0.04), UH Seidman Cancer Center Database (2005–2020).

Regarding risk factors for SCC (Table 3), there was a difference in alcoholism (p = 0.04), with a higher percentage of alcoholics in males (36.7%). There was no difference for Charlson Score (p = 0.58), smoking status (p = 0.49), obesity (p = 1.00), BE (p = 0.92), achalasia (p = 1.00), previous gastrectomy (p = 0.15), gastritis (p = 0.98), gastroesophageal reflux (p = 0.55), H.pylori infection (p = 1.00) and long-term use of NSAIDs (p = 0.83). For treatment characteristics for SCC (Table 4) there was no difference in chemotherapy (p = 1,00), immunotherapy (p = 0.93), radiotherapy (p = 0.39), surgery (p = 0.43), time to chemotherapy (p = 1.00, time to radiation (p = 0.81), time of radiation (p = 1.00), time to surgery (p = 1.00) cisplatin use (p = 0.28), fluorouracil use (p = 0.80), paclitaxel use (p = 0.35), H2 antagonists use (p = 0.85), PPIs use (p = 1.00), NSAIDs use (p = 1.00) and statins use (p = 1.00). For SCC only, the median survival was 17 months for males and 25 months for females (p = 0.80) (Table 5). The univariable analysis did not show inferiority for males (HR = 1.04, CI 0.74–1.46, p = 0.82). The multivariable model included the statistically significant variables (p < 0.20) race, BE, gastritis, gastroesophageal reflux, H.pylori, radiotherapy and surgery. Smoking status and alcoholism were included despite p > 0.20 for being recognized risk factors for this histological subtype. This analysis did not demonstrate inferiority for males (HR = 1.70, CI 0.94–3.07, p = 0.07). The models are summarized on Fig. 3. Sex Differences for SCC are summarized on Fig. 6.

### SEER

Using data from years 2005 to 2018 from SEER (Table 6 and Fig. 2), we analyzed a total of 55,771 patients for all types of esophageal cancer with 77.9% (43,441) males and 22.1% (12,330) females, establishing a male: female ratio of about of 3.5:1. In this cohort, 31,255 had the diagnosis of EAC and 17,540 of SCC, with a predominance of EAC in males (70.5%) and SCC on females (58.7%).

**Table 6:** Charachteristics of esophageal cancer patients from SEER Database diagnosed between 2005–2018.

	SEER								
	All types (n = 55,771)			EAC (n = 31,266)			SCC (n = 17,540)		
	Male	Female	p value	Male	Female	p value	Male	Female	p value
	43,441 (77.9%)	12,330 (22.1%)		26,840 (85.8%)	4426 (14.2%)		11,239 (64.1%)	6301 (35.9%)	
**Age at diagnosis—n (%)**									
18–55 years	5936 (13.7%)	1319 (10.7%)	< 0.001^a^	3812 (14.2%)	511 (11.5%)	< 0.001^a^	1439 (12.8%)	669 (10.6%)	< 0.001^a^
56–70 years	19,538 (45%)	4265 (34.6%)		12,117 (45.1%)	1498 (33.8%)		5153 (45.8%)	2345 (37.2%)	
> 70 years	17,967 (41.4%)	6746 (54.7%)		10,911 (40.7%)	2417 (54.6%)		4647 (41.3%)	3287 (52.2%)	
**Race—n (%)**									
White	37,056 (85.6%)	9774 (79.5%)	< 0.001^a^	25,397 (94.9%)	4055 (92%)	< 0.001^a^	6899 (61.5%)	4374 (69.6%)	< 0.001^a^
Black	4022 (9.3%)	1737 (14.1%)		670 (2.5%)	223 (5.1%)		3003 (26.8%)	1357 (21.6%)	
Other	2235 (5.2%)	777 (6.3%)		694 (2.6%)	132 (3%)		1310 (11.7%)	552 (8.8%)	
Unknown	128	42		79	16		27	18	
**Ethnicity—n (%)**									
Hispanic	3402 (7.8%)	857 (7%)	< 0.001^a^	1884 (7%)	342 (7.7%)	0.09^a^	1083 (9.6%)	381 (6%)	< 0.001^a^
Non-Hispanic	40,039 (92.2%)	11,473 (93%)		24,956 (93%)	4084 (92.3%)		10,156 (90.4%)	5920 (94%)	
Unknown	0	0		0	0				
**Histology—n (%)**									
EAC	26,840 (70.5%)	4426 (41.3%)	< 0.001^a^	–	–	–	–	–	–
SCC	11,239 (29.5%)	6301 (58.7%)		–	–		–	–	
Unknown/Other	5362	1603		–	–		–	–	
**Stage—n (%)**									
I	1939 (5.8%)	575 (6.3%)	< 0.001^a^	1375 (6.3%)	257 (7.3%)	0.20^a^	512 (5.8%)	304 (6.1%)	< 0.001^a^
II	13,450 (40.3%)	4097 (44.9%)		8923 (41%)	1429 (40.4%)		4265 (48.3%)	2620 (52.6%)	
III	17,426 (52.2%)	4297 (47.1%)		11,147 (51.3%)	1802 (50.9%)		3987 (45.1%)	2010 (40.4%)	
IV	547 (1.6%)	163 (1.8%)		292 (1.3%)	49 (1.4%)		68 (0.8%)	47 (0.9%)	
Unknown	10,079	3198		5103	889		2407	1,32	
**Treatment—n (%)**									
Chemotherapy	25,870 (59.6%)	6219 (50.4%)	< 0.001^a^	16,718 (62.3%)	2251 (50.9%)	< 0.001^a^	6709 (59.7%)	3523 (55.9%)	< 0.001^a^
Radiotherapy	22,992 (52.9%)	6124 (49.7%)	< 0.001^a^	14,146 (52.7%)	1970 (44.5%)	< 0.001^a^	6827 (60.7%)	3771 (59.8%)	0.25a
Surgery	10,850 (25%)	2269 (18.4%)	< 0.001^a^	8413 (31.3%)	1103 (24.9%)	< 0.001^a^	1513 (13.5%)	983 (15.6%)	< 0.001^a^
**Vital status—n (%)**									
Alive	8835 (20.3%)	2521 (20.4%)	0.80^a^	6390 (23.8%)	997 (22.5%)	0.06^a^	1782 (15.9%)	1329 (21.1%)	< 0.001^a^
Dead	34,606 (79.7%)	9808 (79.6%)		20,450 (76.2%)	3429 (77.5%)		9457 (84.1%)	4972 (78.9%)	

^a^Chi-square test.

For all types of EC sex differences were found for age at diagnosis (p < 0.001), race (p < 0.001), ethnicity (p < 0.001), histology (p < 0.001), stage (p < 0.001), chemotherapy (p < 0.001), radiotherapy (p < 0.001), surgery (p < 0.001) and median survival (p = 0.03), without differences for vital status (p = 0.80). For EAC there were no differences for ethnicity (p = 0.09), stage (0.20) and vital status (p = 0.06). For SCC there were no differences only for radiotherapy (p = 0.25).

**Figure 7:**
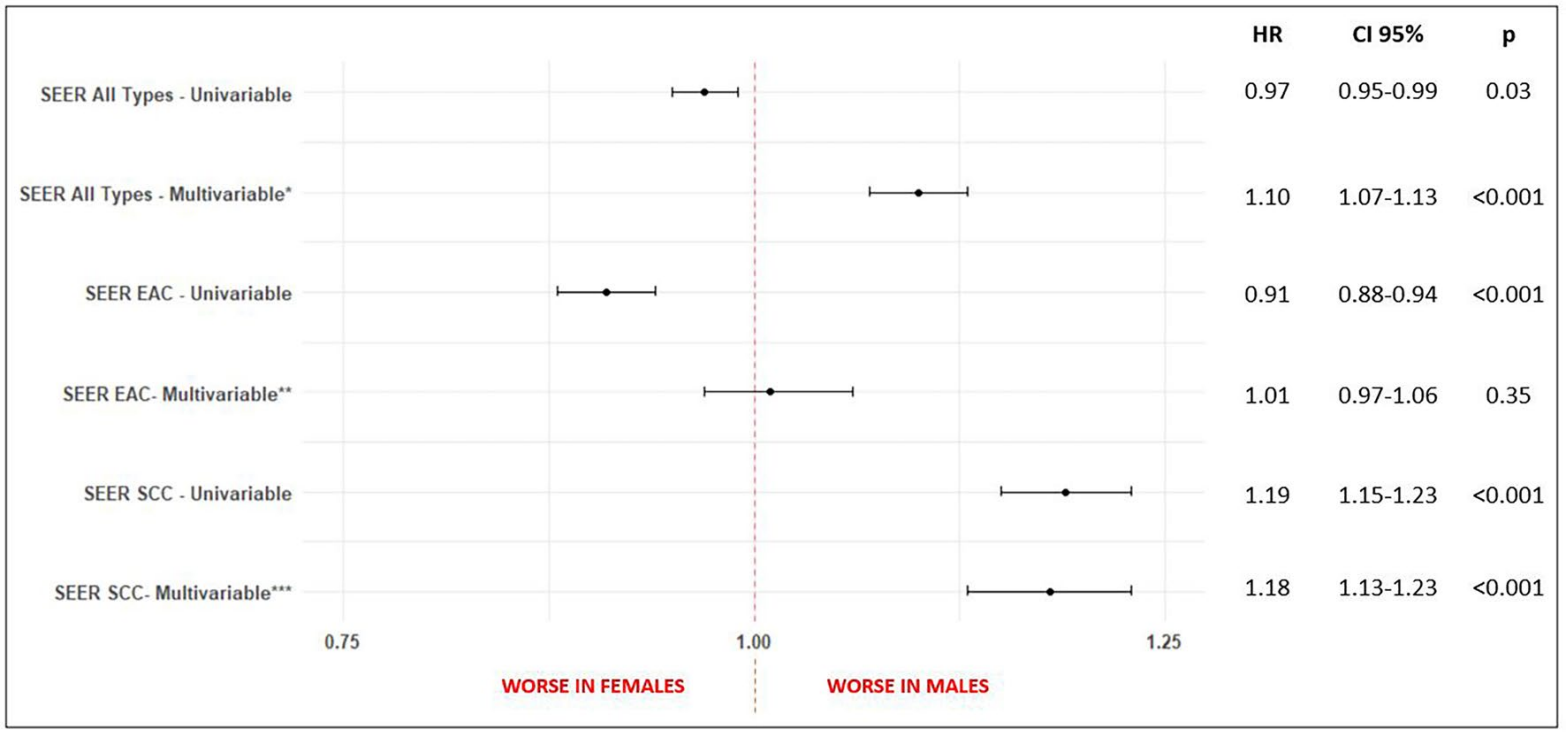
Forest plot of sex differences in survival for esophageal cancer (all types), esophageal adenocarcinoma (EAC) and squamous cell carcinoma (SCC). Univariable and multivariable cox models represented, SEER Database (2005–2018). *Adjusted for: age at diagnosis, race, ethnicity, histology, stage, chemotherapy, radiotherapy, and surgery. **Adjusted for: age at diagnosis, race, ethnicity, stage, chemotherapy, radiotherapy, and surgery. ***Adjusted for: age at diagnosis, race, histology, stage, chemotherapy, radiotherapy, and surgery.

On the Survival Analysis, summarized on Fig. 7, differences were found on all types, EAC and SCC univariable models, while on multivariable EC and SCC showed higher risk of death for males, except on the EAC multivariable (HR for males = 1.01, CI = 0.97–1.06, p = 0.35).

## Discussion

The primary objective of this work was to assess sex differences in a large spectrum of variables and assess the potential effects of these differences on survival for Esophageal Cancer and its two main histological subtypes (Adenocarcinoma—EAC and Squamous Cell Carcinoma—SCC). We believe that our main contribution to the field is the solid, qualified, and detailed information available on our institutional database, that with the integration of disparate sources, enabled us to carry a comprehensive analysis, adding variables and information that helps to understand the epidemiology of sex differences for esophageal cancer.

This study showed that, like other cancers, esophageal cancer and its two main histological subtypes (EAC and SCC) occur more often in males than in females, on both our institutional database and SEER, corroborating with literature reports of higher incidence in males^38,35^. The mechanisms to explain these sex differences are not fully understood and seems to be multifactorial, mainly involving hormonal and genomic factors^38,36^. Our study also showed that, regarding risk factors, there are differences only on smoking status and only when analyzing all types of esophageal cancer (probably due to the inclusion on other/unknown histology diagnosis on this group), in line with findings that risk factors doesn’t seem to be associated with the higher incidence in males^37,38^. Looking to demographic factors we noted that there are differences for age at diagnosis for all types of Esophageal Cancer and for SCC, with females tending to be diagnosed at older ages, findings that can contribute to the hypothesis that estrogen can be an inhibitor for the esophageal carcinogenesis and thus protective for females on the pre-menopausal stage^38–40^.

Regarding cancer characteristics (Table 2) the only difference seen is on the histological subtype. Besides our institutional database confirming the trends of higher rates of EAC for both sexes, it interestingly also showed that females have 2 × higher rates of SCC than males and lower rates of EAC than their counterparts (while 43.5% of the females have SCC diagnosis, only 20.8% of males have this diagnosis), while SEER showed a predominancy on SCC in females (58.7%). Since the female diagnosis of this subtype of cancer is predominantly at post-menopausal ages this could indicate that the estrogen protective effect is higher on the SCC subtype, corroborating with some previous studies^41^.

Excluding the differences already mentioned on age at diagnosis, smoking status and histological subtype, the only other differences between males and females are on alcoholism (only for SCC) and NSAIDs prescription (only for all types of Esophageal Cancer). All the other various variables analyzed didn’t showed any sex differences. Smoking status and alcoholism differences seems to be explained by populational behavior differences and, together with the other differences found, don’t seem to be to have an impact on the outcomes. Regarding to the outcomes, there’s not statistical significance but it’s possible to see a tendency of higher hazard ratios for males. The literature is conflicting about sex differences on survival, while some studies report worse outcomes in males, other report no differences, just like our study^19,20,22,42–44^. In addition, we observed changes in diagnosis over time (Fig. 2), with an increasing trend in Esophageal Cancer overall, with increasing number of EAC cases and a downward trend in the number of cases of SCC, especially in male patients. These trends corroborate findings in the literature regarding expectations of an increase in incidence of Esophageal Cancer, especially EAC, on US and an decrease in SCC, probably associated with a reduction in alcohol and tobacco consumption^17,45^.

Interestingly SEER data showed different patterns from our population. These differences reported between our database and SEER reflects differences in quality of care and population treated inside the US, for example with the underrepresentation of Hispanic patients and higher rates of treatment on our population. Our institution is localized on the state of Ohio, that, accordingly to the Ohio Department of Health, has an average of 781 new esophageal cancer cases per year, with an incidence rate of 5.2 per 100,000 (number 23% higher than the US rate) and annual cases of 625 per 100,000 for males vs 156 cases per 100,000 for females and all providers are required, by law, to report to Ohio Cancer Incidence Surveillance System (OCISS) all cancers diagnosed and/or treated on the state.

This study has several limitations. Our institutional database is based on the specific population being followed up on the University Hospitals Seidman Cancer Center, thus our selection does not indicate a population sample outside this context. Since this service also receives patients already diagnosed and being treated on other services, some of the information on the EMR may be incomplete. In addition, given the retrospective nature of this study, some valuable variables (such as the location of the cancer on the esophagus) are not available and for some variables there is a high number of NAs/unknown information (such as histological subtype and clinical staging) and this missing information can lead to a loss of statistical power. Also, the median income variable was generated by patient`s zip code and thus could have some misclassification. On the other hand, we analyzed a high number of variables with detailed information, giving new insights for what`s already published on the field. Additional studies with other databases, larger cohorts and with prospective design are needed to corroborate and investigate the findings reported here.

In summary, we found that males have a higher incidence of Esophageal Cancer and its two main subtypes (EAC and SCC) but none of the comprehensive set of variables analyzed showed to be strongly or unique correlated with this sex difference in incidence nor are they associated with a sex difference in survival.

## Supplementary Information

Supplementary Table 1.

## Data Availability

University Hospitals (UH) Seidman Cancer Center database is available at University Hospitals Cleveland Medical Center and have access restricted to researchers with IRB approval. SEER database is an US open-access database from National Cancer Institute (NCI/NIH) and can be accessed at https://seer.cancer.gov/data/access.html.
