# Supplementary material for: Sex differences in esophageal cancer overall and by histological subtype
Source: Sci Rep. 2022 Mar 28;12:5248. doi: 10.1038/s41598-022-09193-x (PMC8960903; doi:10.1038/s41598-022-09193-x)
Supplement: Supplementary file 1 — Supplementary Table 1. [file 41598_2022_9193_MOESM1_ESM.docx]

**Supplementary Data**

**Supplementary Table 1.** Categorization process for UH Seidman Cancer Center Database.

| **Variable** | **Type (raw)** | **Categorization** | **Main Classification** |
| --- | --- | --- | --- |
| Age at diagnosis | Continuous | 18-55 years | General characteristics |
|  |  | 56-70 years |  |
|  |  | > 70 years |  |
| Race | Categorical | White |  |
|  |  | Black |  |
|  |  | Other |  |
| Ethnicity | Categorical | Hispanic |  |
|  |  | Non-hispanic |  |
|  |  | Other |  |
| Median Income | Categorical (zip code) | <$40,227 |  |
|  |  | $40,227-$50,353 |  |
|  |  | $50,354-$63,332 |  |
|  |  | >$63,333 |  |
| Histology | Categorical | SCC (ICD-O-3 8050-8084) | Cancer Characteristics |
|  |  | EAC (ICD-O-3 8140-8384) |  |
| Clinical/Pathological Staging | Categorical | I |  |
|  |  | II |  |
|  |  | III |  |
|  |  | IV |  |
| Charlson Comorbidity Score | Categorical (list of comorbidities) | 0 |  |
|  |  | 1 to 2 |  |
|  |  | 3 to 4 |  |
|  |  | >=5 |  |
| Smoking Status | Categorical | Yes | Risk Factors |
|  |  | No |  |
|  |  | Former |  |
| Additional Known Risk Factors | Categorical (list of comorbidities) | Obesity (y/n) |  |
|  | Categorical (list of comorbidities) | Barret's (y/n) |  |
|  | Categorical (list of comorbidities) | Alcoholism (y/n) |  |
|  | Categorical (list of comorbidities) | Achalasia (y/n) |  |
|  | Categorical (list of comorbidities) | Previous Gastrectomy (y/n) |  |
|  | Categorical (list of comorbidities) | Gastritis (y/n) |  |
|  | Categorical (list of comorbidities) | Gastroesophageal Reflux (y/n) |  |
|  | Categorical (list of comorbidities) | H.Pylori Infection (y/n) |  |
|  | Categorical (list of comorbidities) | Long term use of NSAIDs (y/n) |  |
| Type of Treatment | Categorical | Immunotherapy (y/n) | General Treatment |
|  | Categorical | Chemotherapy (y/n) |  |
| Radiation | Categorical (list of all radiation) | Radiation (y/n) |  |
| Surgeries | Categorical (list of all surgeries) | Surgery (y/n) |  |
| Time to Chemotherapy | Continuous | <40 days |  |
|  |  | >= 40 days |  |
| Time to Radiation | Continuous | <40 days |  |
|  |  | >= 40 days |  |
| Time of Radiation | Continuous | <40 days |  |
|  |  | >= 40 days |  |
| Time to Surgery | Continuous | <40 days |  |
|  |  | >= 40 days |  |
| Medications Prescribed | Categorical (list of all medications) | Cisplatin (y/n) |  |
|  | Categorical (list of all medications) | Fluorouracil (y/n) |  |
|  | Categorical (list of all medications) | Paclitaxel (y/n) |  |
|  | Categorical (list of all medications) | H2 Antagonists (y/n) |  |
|  | Categorical (list of all medications) | PPIs (y/n) |  |
|  | Categorical (list of all medications) | NSAIDs (y/n) |  |
|  | Categorical (list of all medications) | Statins (y/n) |  |
| Vital Status | Categorical (date of death) | Dead (y/n) | Outcomes |
